# Radiation Segmentectomy for the Treatment of Hepatocellular Carcinoma: A Practical Review of Evidence

**DOI:** 10.3390/cancers16030669

**Published:** 2024-02-04

**Authors:** Sophia N. Mourad, Cynthia De la Garza-Ramos, Beau B. Toskich

**Affiliations:** 1 College of Medicine, Florida State University, Orlando, FL 32301, USA; 2 Division of Interventional Radiology, Mayo Clinic Florida, Jacksonville, FL 32224, USA

**Keywords:** Y90, radioembolization, radiation segmentectomy, hepatocellular carcinoma

## Abstract

**Simple Summary:**

This article is an overview of the technique, indications, and outcomes of transarterial yttrium-90 radiation segmentectomy for the treatment of hepatocellular carcinoma (HCC) and is intended to provide a pragmatic summary for any member of a hepatobiliary malignancy multidisciplinary team.

**Abstract:**

Radiation segmentectomy is a versatile, safe, and effective ablative therapy for early-stage hepatocellular carcinoma. Advances in radiation segmentectomy patient selection, procedural technique, and dosimetry have positioned this modality as a curative-intent and guideline-supported treatment for patients with solitary HCC. This review describes key radiation segmentectomy concepts and summarizes the existing literary knowledgebase.

## 1. Introduction

Hepatocellular carcinoma (HCC) is the most common primary hepatic malignancy and is associated with high morbidity and mortality rates worldwide. While liver transplantation, surgical resection, and thermal ablation are considered first-line therapies for early-stage HCC, many patients are not candidates for these interventions due to underlying liver disease, tumor location, and comorbidities. In these circumstances, alternative locoregional therapies such as transarterial radioembolization (TARE) or chemoembolization (TACE) are recommended by multiple international guidelines [1,2,3].

TARE consists of the administration of radioactive microspheres into the blood vessels supplying the tumor, with the goal of devitalizing tumor tissue via microscopic brachytherapy. Advancements in technique and dosimetry have broadened the use of radioembolization within the HCC spectrum ranging from very early- to advanced-stage disease. TARE can be offered with palliative intent, as a neoadjuvant to resection or liver transplantation, or as definitive therapy.

Radiation segmentectomy, also known as ablative radioembolization, involves the selective delivery of high-dose radiation (a perfused volume dose > 400 Gy) to two Couinaud hepatic segments or less with the goal of complete tumor obliteration [4,5,6,7]. Multiple studies have shown that radiation segmentectomy outcomes for early-stage HCC are comparable to other curative therapies, despite being applied to patients with more challenging disease presentations [8,9].

The multicenter, retrospective, LEGACY study established ablative radioembolization as a safe and effective treatment option for patients with solitary, unresectable HCC ≤ 8 cm [10]. As a result, radioembolization with glass microspheres received United States Food and Drug Administration (FDA) approval and was included in the Barcelona Clinic Liver Cancer (BCLC) guidelines as a treatment for patients with solitary tumors ≤8 cm with preserved liver function who are not candidates for or have failed resection or thermal ablation [1]. The National Comprehensive Cancer Network guidelines acknowledge the use of radiation segmentectomy and its recommended dosimetry for patients with small HCC, adequate liver function, and tumor characteristics that are amenable to this treatment approach [2]. The American Association for the Study of Liver Diseases recommends radiation segmentectomy as an alternative ablative therapy to thermal ablation for BCLC A patients [3].

These advancements have solidified the place for radiation segmentectomy within the current HCC treatment armamentarium. The aim of this literature review is to provide an overview of radiation segmentectomy technicalities, summarize outcomes reported in the literature, and identify areas for future investigation.

## 2. Technique and Dosimetry

The most common isotope used in transarterial radioembolization is Yttrium-90 (Y90), an almost pure beta-particle emitter that exerts tumoricidal effects via radiation-induced DNA damage-associated cell death. The two most widely studied Y90-containing radioembolization devices are glass microspheres (TheraSphere; Boston Scientific, Marlborough, MA, USA) and resin microspheres (SIR-Spheres; Sirtex Medical Inc., Woburn, MA, USA). Glass microspheres have FDA approval for solitary, unresectable HCC in patients with preserved liver function, and resin microspheres have FDA approval for the treatment of colorectal metastases to the liver with concurrent intraarterial floxuridine [11,12].

Compared to glass, resin microspheres have a lower specific activity (activity per microsphere) and specific gravity that could contribute to differences in radiobiology in addition to intravascular transport and intratumor dissemination [13,14]. The total number and distribution of microspheres per treatment volume and the specific activity dictates the overall patient dose. There are limited studies comparing glass vs. resin microspheres [15,16]; however, the majority of the radiation segmentectomy literature is based on glass microspheres and will hence be the focus of this review.

The FDA-approved and expert consensus-endorsed dosimetry methodology for Y90-containing glass microsphere radiation segmentectomy is the single-compartment Medical Internal Radiation Dose (MIRD) schema [17]. Although the MIRD methodology erroneously assumes equal distribution of particles across the perfused treatment volume, known as an angiosome, it has been shown to be safe, effective, and reproducible. Other methods, such as multi-compartment and 3D-voxel dosimetry, have not been studied as well for radiation segmentectomy and rely on pretreatment Technetium-99m macroaggregated albumin (99mTc-MAA) simulation, which is an inconsistent surrogate for smaller tumors [18].

Radioembolization is an outpatient procedure that is typically performed in two stages. First, mapping angiography and contrast-enhanced cone-beam computed tomography are performed to identify tumor-supplying arteries and quantify the treatment angiosome coverage and volume [19]. Transarterial infusion of 99mTc-MAA as a microsphere surrogate, followed by SPECT/CT are used to approximate particle deposition and calculation of the lung shunt fraction (LSF) to assess the risk of radiation injury to the lung, namely in larger tumors. The radiation dose safety threshold for the lungs has been historically set as no more than 30 Gy in one session or 50 Gy in a lifetime, according to external beam radiotherapy and historical radioembolization data [20]. LSF calculation using the planar technique, although commonly used, has been found to overestimate the true LSF and potentially lead to inappropriate dose reduction or procedure cancellation [21]. For patients undergoing radiation segmentectomy who typically have a low LSF, a more accurate calculation with SPECT is unlikely to be clinically significant [21].

**Figure 1:**
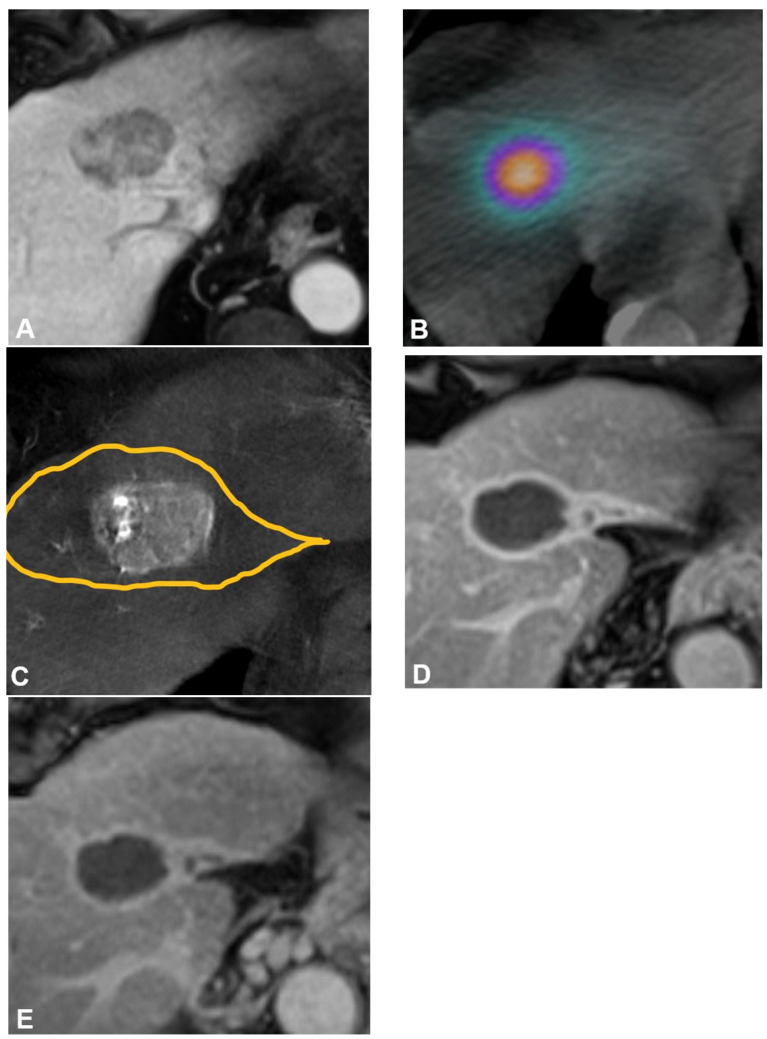
Radiation segmentectomy as sole therapy for hepatocellular carcinoma in a patient with a history of hepatitis C virus, stage 4 fibrosis, and ALBI 1 and Child-Pugh A5 liver function. (**A**) Pretreatment axial contrast-enhanced MRI demonstrates a 4.3 cm tumor in hepatic segment Iva. (**B**) Cone-beam CT performed at the time of mapping angiography demonstrates the arterially enhancing target tumor is fed by a segment IVa hepatic artery. The yellow line delineates the estimated treatment angiosome. (**C**) Posttreatment axial SPECT/CT confirms activity within the targeted angiosome and absent extrahepatic deposition. The estimated radiation dose was 870 Gy MIRD. Follow-up axial contrast-enhanced MRI obtained (**D**) 12 months and (**E**) 52 months after therapy demonstrate no evidence of residual or recurrent disease, and contraction of the treated angiosome.

Treatment commonly occurs one to four weeks after mapping and consists of selective transarterial infusion of Y90-containing microspheres within target vessels followed by Bremsstrahlung SPECT/CT or Y90 PET/CT to confirm particle deposition (Figure 1). To enhance dose delivery to the tumor and reduce other nontarget uptake, flow diversion prior to radiation segmentectomy using coils, balloon occlusion, gel foam, and plugs can be implemented when indicated [22,23,24]. Same-day mapping and treatment are now being performed more often to expedite oncologic care [25,26,27]. Recent studies suggest that pretreatment 99mTc-MAA be omitted in patients with early-stage HCC due to the low LSF and risk of radiation pneumonitis in patients with these baseline tumor characteristics and absence of a transjugular intrahepatic portosystemic shunt [28].

## 3. Indications and Patient Selection

Radiation segmentectomy is most commonly utilized for patients with very-early to early-stage BCLC 0-A disease and preserved liver function (Child-Pugh A to B7), who are ineligible for or have failed surgical resection and/or thermal ablation. In patients with potentially resectable tumors without an appropriate estimated liver remnant volume, combining a radiation segmentectomy approach to the tumor with a “radiation lobectomy” dose to the future resection site can be used as neoadjuvant therapy to induce hypertrophy of the future liver remnant [29,30]. Patients with BCLC B-C disease or Child-Pugh B7-C liver function can be considered for radioembolization as a definitive therapy or as a bridging or downstaging to a transplant approach [1,2,3]. Patient selection should be based on individual evaluation and discussion by a multidisciplinary team.

Radiation segmentectomy can be performed in patients with newly diagnosed HCC as well as patients with prior locoregional therapy as long as the vascular anatomy is favorable [17]. Compared to thermal ablation, radiation segmentectomy can be performed in proximity to surrounding critical structures with a very low risk of adverse events and, unlike thermal ablation, efficacy rates are not limited to tumors < 3 cm [7,31].

## 4. Imaging Response Assessment

Initial follow-up imaging with abdominal multiphase contrast-enhanced magnetic resonance imaging or contrast-enhanced computed tomography is typically performed at one, three, and six months posttreatment. As radioembolization exerts gradual tissue devitalization, the maximum tumor response is often observed several months after treatment [32]. Imaging response to radiation segmentectomy appears to occur earlier (within one to three months) than conventional radioembolization (three to six months) [33].

While imaging response for solid tumors is often assessed as a function of tumor size reduction, HCC response to ablative radioembolization is more accurately assessed by degree of tumor enhancement as a marker of tumor viability; therefore, the modified Evaluation Criteria in Solid Tumors (mRECIST) or European Association for the Study of the Liver (EASL) are the preferred criteria. Complete resolution of arterial enhancement indicates complete necrosis by mRECIST criteria [34]. Fibrosis and retraction of the hepatic capsule around the treatment site, with hypertrophy of the hepatic remnant, can be seen in later imaging and is associated with improved pathologic response rates [35]. A small radiopathologic series that aimed to assess for imaging surrogates of histologic response found a correlation between complete pathologic response and the absence of hepatocyte-specific contrast uptake, hyperintensity on T2-weighted sequences, and plateau or persistent enhancement in the treatment angiosome on posttreatment MRI [36].

## 5. Pathologic Response and Associated Treatment Parameters

Multiple radiopathologic studies on Y90-containing glass microsphere radioembolization for HCC have shown a positive correlation between tumor radiation dose and pathological response, or degree of tumor necrosis, on histopathological examination after liver transplantation or hepatectomy.

The historical ablative dose threshold of >190 Gy was established from the first radiation segmentectomy radiopathologic analysis by Vouche et al. on 33 patients, where 67% (14/21) of those treated with a dose > 190 Gy MIRD had complete pathologic necrosis (CPN) [5]. Subsequently, on an explant analysis of 45 patients from the LEGACY cohort, Gabr et al. reported that while 86% of patients receiving doses > 190 Gy achieved CPN, 100% of patients treated with a dose > 400 Gy achieved CPN [6]. In a validation study, Toskich et al. supported the importance of higher tumor doses, with a CPN rate of 53% with doses > 190 Gy and 75% with >500 Gy [7].

Most recently, it has been shown that other treatment parameters beyond dose are associated with pathologic response. Glass microsphere specific activity corresponding to first week administration or up to Monday of the second week (≤8-day decay from calibration) has been described as an independent predictor of CPN [7,31]. In a large, single-center radiopathologic study of 75 tumors, Montazeri et al. compared a baseline cohort treated with a wide range of treatment parameters to a treatment intensification cohort that received higher doses and specific activities (≥400 Gy and ≤8-day decay from calibration, respectively) and reported a significantly higher rate of CPN in the treatment intensified cohort (76% vs. 49%) [31]. This study emphasized that both specific activity and dose should be prioritized to achieve the best outcomes.

## 6. Outcomes

### Efficacy

In the earliest study of radiation segmentectomy in 2011, Riaz et al. demonstrated a treatment response of 81% using the EASL criteria, a median time-to-progression (TTP) of 13.6 months, and median survival of 26.9 months [4]. In 2014, Vouche et al. conducted a multicenter study of 102 patients with unresectable HCC ≤ 5 cm and found an 87% objective response rate (ORR) by using the mRECIST criteria, defined as complete or partial response, a median TTP of 33.1 months, and median overall survival (OS) of 53.4 months [5]. It was also found that certain patient characteristics, namely age < 65 years, ECOG 0, and Child-Pugh A, were associated with increased survival [5].

A long-term outcome analysis by Lewandowski et al. of 70 patients with HCC ≤ 5 cm and preserved liver function reported an ORR of 86% and 49% at 6 months using EASL and WHO criteria, respectively; a median TTP of 2.4 years with 72% of patients having no target lesion progression at 5 years; and a median OS of 6.7 years [8]. The retrospective LEGACY study for patients with solitary, unresectable HCC ≤ 8 cm, ECOG 0–1, and Child-Pugh A liver function reported an ORR of 88.3%, a duration of response ≥6 months in 62.2% of patients, and a three-year OS of 86.6% for all patients and 92.8% for patients who subsequently underwent surgical resection or liver transplantation [10].

The phase II, prospective, single-arm RASER trial evaluated the curative efficacy of radiation segmentectomy in 29 patients with HCC ≤ 3 cm in suboptimal locations for percutaneous ablation, ECOG 0, and Child-Pugh A-B7 liver function, and found that all patients had an initial objective response by using mRECIST (complete: 83%; partial: 17%) and 90% (*n* = 26) had a sustained objective response at a median follow-up of 691 days (IQR 379–719) [37]. Eight patients underwent subsequent liver transplantation and all (*n* = 8/8) target lesions exhibited CPN [37].

## 7. Comparison to Other Locoregional Therapies

Two historical phase II randomized controlled trials, PREMIERE and TRACE, compared the effects of TARE vs. conventional TACE and drug-eluting bead TACE, respectively; both trials reported significantly superior TTP in the TARE cohorts [38,39]. The TRACE trial also reported superior survival in the TARE cohort compared to drug-eluting bead TACE (median OS 30.2 months vs. 15.6 months, *p* = 0.006); surprisingly, TTP in the TARE arm (median 17.1 months) was superior to the median survival in the TACE arm [39].

Similarly, multiple studies have shown the benefits of radiation segmentectomy compared to other available locoregional therapies. In a propensity-score matched retrospective study of radiation segmentectomy vs. segmental chemoembolization for 235 tumors by Padia et al., radiation segmentectomy demonstrated higher complete response rates (92% vs. 74%, *p* = 0.001), lower target tumor progression at one year (8% vs. 30%, *p* < 0.001) and two years (15% vs. 42%, *p* < 0.001), and a longer median progression-free survival (PFS) with and without censoring for liver transplantation, with similar toxicity profiles [40]. In a subsequent propensity score-matched retrospective study of radiation segmentectomy compared to segmental chemoembolization for solitary HCC ≤ 3 cm, Biederman et al. similarly found higher complete response rates with radiation segmentectomy (92% vs. 53%, *p* = 0.005), as well as longer times to secondary therapy (812 vs. 161 days, *p* = 0.001) [41].

When compared to a combination chemoembolization and microwave ablation regimen for patients with unresectable, solitary HCC ≤ 3 cm, radiation segmentectomy achieved similar overall complete response rates, median TTP, and OS [42]. In a single-center study of treatment-naïve patients with HCC ≤ 4 cm treated with radiation segmentectomy or microwave ablation alone, the radiation segmentectomy cohort exhibited a longer target tumor mean PFS (57.8 vs. 38.6 months, *p* = 0.005), with a similar safety profile, tumor response rates, overall progression, and OS [43]. These studies support the use of radiation segmentectomy as a standalone ablative modality, particularly in cases not suitable for thermal ablation due to tumor location or size.

Notably, radiation segmentectomy outcomes have, thus far, been comparable to surgical resection for early-stage HCC, with a significantly lower incidence of major adverse events (AE). A retrospective cohort study of 123 treatment-naïve patients with solitary HCC ≤ 8 cm who underwent either radiation segmentectomy or surgical resection found similar rates of target tumor and overall progression [9]. Although overall TTP was longer in those treated with resection (29 vs. 22 months, *p* = 0.003), when the cohorts were analyzed by factors known to be associated with disease recurrence such as thrombocytopenia and advanced liver fibrosis, overall TTP did not differ between treatment groups [9]. This study also highlighted how the patient population that undergoes radiation segmentectomy is intrinsically different to those considered good surgical candidates. An additional study comparing TARE to surgical resection as an initial treatment for large (≥5 cm) HCC (including patients with minute satellite lesions or tumor thrombosis involving minor portal vein branches) found similar intrahepatic TTP, overall TTP, and OS between cohorts after inverse-probability-of-treatment weighting [44]. Table 1 summarizes the imaging and survival outcomes of radiation segmentectomy compared to other available locoregional therapies.

## 8. Safety

Radiation segmentectomy may be associated with mild, often transient AE. Fatigue, fever, nausea, vomiting, anorexia, and abdominal discomfort are the most common clinical symptoms after treatment. At imaging follow-up, small-volume, localized ascites around the treatment site can be visualized but tend to be clinically insignificant. Laboratory and biochemical AE include thrombocytopenia, lymphopenia, increased alkaline phosphatase, increased aspartate or alanine aminotransferase, and decreased albumin [45]. Major biochemical AE are uncommon and have been reported in up to 25% of patients, with silent lymphopenia being the most frequent AE [46,47]. Radioembolization-induced liver disease and radiation pneumonitis, potential significant AEs with conventional whole liver or bilobar radioembolization, have not been reported in the radiation segmentectomy literature [10,37,45,47].

Patients with altered liver function prior to treatment and large volumes of liver being treated are at greater risk of AE. De la Garza-Ramos et al. attempted to identify a threshold for the amount of liver that can be treated without significant biochemical AE with radioembolization using glass microspheres and a dose > 190 Gy MIRD [47]. A percent liver treated ≥ 14.5% was associated with a higher risk of AE in patients with a baseline ALBI 2 or Child-Pugh B liver function. Additionally, a baseline whole liver volume < 1.3 L was reported to be an independent factor in the development of grade 2 albumin or bilirubin AE [47]. This study does not imply these patients should not undergo treatment, but rather guides risk stratification and informed decision making.

The shift in the ablative tumor dose threshold and expansion of interventional radiologists’ knowledge of radiation segmentectomy has led to treatment with higher MIRD doses. A small case series analyzed the safety of radiation segmentectomy with doses > 1000 Gy MIRD in 11 patients with solitary HCC and demonstrated similar safety and efficacy outcomes as previously published studies, with no increase in incidence of AE [33]. While there have been isolated reports that suggest a theoretical radiation risk to adjacent organs abutting tumors with high-dose radiation segmentectomy, this has not been reported in larger studies [48]. Although there is no defined dosimetry upper limit, this study has provided a foundation to suggest that high dose radiation segmentectomy is equally as safe, given the low volumes of liver being treated and the high selectivity of this technique.

## 9. Future Directions

In the past two decades, advances in radiation segmentectomy technique and dosimetry have allowed the establishment of this modality as an FDA-approved and guideline-endorsed treatment for solitary, early-stage HCC. A particularly growing future direction is the study of combination radioembolization and immunotherapy [49,50]. While there is evidence to suggest a potential difference in response to immunotherapy based on nonviral vs. viral-related HCC, a recent retrospective study on radiation segmentectomy in nonalcoholic fatty liver disease vs. hepatitis C virus-related HCC demonstrated comparable outcomes, suggesting that response to therapy would not be dictated by etiology of liver disease [51].

Multiple ongoing clinical trials are investigating the potential synergistic effect of immunotherapy and ablative radioembolization, and whether this translates into therapeutic benefit remains to be established. A phase I/IIa study on the combination of radioembolization and subsequent intravenous durvalumab in patients with locally advanced HCC showed promising results with a median TTP of 15.2 months and an objective response rate of 83%, with a low rate of grade 3 AE (9%) [52]. The role of ablative radioembolization in the setting of immunotherapy for patients with limited vascular invasion is also under current investigation (NCT05063565).

## 10. Conclusions

Radiation segmentectomy is now established as a versatile ablative treatment for patients with early-stage hepatocellular carcinoma. Its competitive outcomes in treating tumors that are not candidates for thermal ablation or resection make it an indispensable therapy in current HCC care. Radiation segmentectomy should be used to augment definitive therapy options for patients with HCC as part of a comprehensive multidisciplinary program.

## Figures and Tables

**Table 1:** Summary of imaging and survival outcomes in comparative studies.

Author	Therapy	Cohort Size	PSM	Imaging Outcomes	Progression Outcomes	Survival Outcomes
Padia et al., 2017 [40]	RS	*n* = 101	Yes	OR 94% by mRECIST *	ITP1 7.7% ITP2 15%	OS 1198 d
	TACE	*n* = 77		OR 84% by mRECIST *	ITP1 30% ITP2 42%	OS 1043 d
Biederman et al., 2018 [41]	RS	*n* = 55	Yes	CR 94.7% *	TTST 812 d *	OS 27.6 m
	TACE	*n* = 57		CR 47.4% *	TTST 161 d *	OS 27.4 m
Biederman et al., 2017 [42]	RS	*n* = 41	Yes	CR 85% by mRECIST	TTP 11.1 m	OS 30.8 m
	TACE + MWA	*n* = 80		CR 85% by mRECIST	TTP 11.6 m	OS 42.7 m
Arndt et al., 2021 [43]	RS	*n* = 34	Yes	OR 90.9% by mRECIST	Target tumor PFS not reached * Overall PFS not reached	OS not reached
	MWA	*n* = 34		OR 82.6% by mRECIST	Target tumor PFS 58.1 m * Overall PFS 28.9 m	OS 58.0 m
De la Garza-Ramos et al., 2022 [9]	RS	*n* = 57	No	OR 98% by mRECIST	Target tumor TTP not reached Overall TTP 21.9 m *	OS not reached
	Surgery	*n* = 66		N/A	Target tumor TTP not reached Overall TTP 29.4 m *	OS not reached

* Statistically significant difference. PSM: propensity score-matched, TACE: transarterial chemoembolization, TARE: transarterial radioembolization, OR: objective response rate, EASL: European Association for Study of the Liver, TTP: time to progression, OS: overall survival, RS: radiation segmentectomy, mRECIST: modified Response Evaluation Criteria in Solid Tumors, ITP1: index tumor progression at one year, ITP2: index tumor progression at two years, d: days; m: months; CR: complete response, TTST: time to secondary therapy, MWA: microwave ablation, PFS: progression-free survival, N/A: not applicable.

## Abbreviations

HCC	hepatocellular carcinoma
TARE	transarterial radioembolization
TACE	transarterial chemoembolization
FDA	Food and Drug Administration
BCLC	Barcelona Clinic Liver Cancer
Y90	Yttrium-90
MIRD	Medical Internal Radiation Dose
99mTc-MAA	Technetium-99m macroaggregated albumin
LSF	lung shunt fraction
mRECIST	modified Evaluation Criteria in Solid Tumors
EASL	European Association for the Study of the Liver
CPN	complete pathologic necrosis
TTP	time-to-progression
OOR	objective response rate
OS	overall survival
PFS	progression free survival
AE	adverse events
